# Kinetics of inflammatory markers following cancer-related bowel and liver resection

**DOI:** 10.3109/03009734.2010.519446

**Published:** 2011-04-12

**Authors:** Sándor Márton, János Garai, Valéria Molnár, Vera Juhász, Lajos Bogár, Tamás Köszegi, Boglárka Falusi, Subhamay Ghosh

**Affiliations:** ^1^University of Pécs, Department of Anaesthesiology and Intensive Therapy, Pécs, Hungary; ^2^University of Pécs, Department of Pathophysiology and Gerontology, Pécs, Hungary; ^3^University of Pécs, Department of Clinical Chemistry, Pécs, Hungary

**Keywords:** C-reactive protein, endotoxin, macrophage migration inhibitory factor, procalcitonin, tumour necrosis factor

## Abstract

**Background:**

Macrophage migration inhibitory factor (MIF) was originally described as a cytokine that inhibits migration of macrophages at the site of inflammation. Subsequently it was also identified as a stress-induced hormone released from the anterior pituitary lobe in response to some pro-inflammatory stimuli like endotoxins and tumour necrosis factor (TNF-α).

**Aim:**

To compare postoperative changes in serum MIF levels of patients undergoing bowel and liver resections. It has clinical relevance to describe the kinetics of this crucial mediator of systemic inflammation in surgery.

**Methods:**

A total of 58 patients were studied over 4 years. Group A (28 patients) underwent only hepatic resection without enterotomy. Group B (30 patients) had bowel resection with enterotomy. MIF, IL-1β, IL-8, prealbumin, albumin, α1-glycoprotein, fibrinogen, and C-reactive protein levels were measured preoperatively, immediately following surgery, and postoperatively for three consecutive days. To evaluate organ functions, multiple organ dysfunction score was used.

**Results:**

A significantly higher level of MIF (4,505 pg/mL) was found in group A when compared to that of group B immediately following surgery. Other parameters monitored in this study were not statistically different between the two groups.

**Conclusion:**

Higher elevations in MIF levels with liver resections, compared to bowel resections, might be attributable to MIF release from damaged liver cells. The presumably minimal endotoxin exposure during bowel surgery was either insufficient or inefficient to induce relevant MIF elevations in our patients. To fully delineate implications of this finding further studies are needed.

## Introduction

Macrophage migration inhibitory factor (MIF) was the first representative of potent immunomediator cytokines that have been described from the beginning of the 1960s (1). Its release from activated T lymphocytes was first considered as its sole source, and its action was thought to be limited to prevention of macrophage migration away from the site of inflammation. MIF is now considered to be a stress-induced cytokine hormone produced by several immune cell types, as well as by the anterior pituitary gland, and its most important role is in the counter-regulation of the anti-inflammatory effects of glucocorticoids (2).

The stress-induced MIF secretion from the adenohypophysis is primarily increased by pro-inflammatory stimuli, i.e. endotoxin, tumour necrosis factor, and also by glucocorticoids (3).

*In-vivo* models have shown that the level of hypophyseal MIF fell significantly after intraperitoneal lipopolysaccharide injection accompanied by an increase of MIF mRNA synthesis and raised MIF levels in the serum (4). An interesting study showed that endotoxin-related mortality was increased by MIF in mice, while administration of anti-MIF antibodies revealed the contrary (5). In hypophysectomized mice Calandra et al. attributed bacterial infection-induced MIF production primarily to macrophages (6). A systematic analysis of MIF expression following administration of lipopolysaccharide revealed that within 6 hours of bacterial inoculation MIF production increased not only in the pituitary and the macrophages, but also in the lungs, adrenal glands, spleen, and the liver of rats (7).

Clinical studies have shown that MIF has a primary role in endotoxaemia-induced toxic reactions, in sepsis (8), and acute respiratory distress syndrome (ARDS) (9). In critically ill patients, suffering from septic shock, an increased level of MIF was found, but this was lower in the survivors compared to the non-survivors (10). Surgical trauma is associated with an increased level of adrenocorticotropic hormone (ACTH) and cortisol (11). In patients suffering from blunt trauma MIF levels were higher in the non-survivor than in the survivor groups (12). Even higher serum levels of MIF were found by Satoshi et al. in patients undergoing liver resection for treatment of hepatic neoplasm (13).

In our prospective, descriptive clinical study we investigated the changes of MIF levels and other indicators of systemic inflammation in patients undergoing bowel resection with presumably higher endotoxin levels (14,15), compared with patients having hepatic resection without enterotomy. Based on our hypothesis, we expected a significant increase in MIF levels in both groups. Following that, we correlated it with early postoperative morbidity and mortality.

## Methods

### Patients

Following the approval of the local ethical committee, 58 patients were studied between October 2004 and December 2008. Group A included cancer patients who had liver resection without enterotomy (28 patients). In group B, patients underwent bowel resection with enterotomy (30 patients). Based on our exclusion criteria, patients undergoing emergency operations without bowel preparation, inoperable carcinoma, and patients with chronic organ failure based on acute physiology and chronic health evaluation (APACHE II) scores were excluded from the investigation.

### Methods

In all the patients, anaesthesia was induced with propofol (2 mg/kg) and fentanyl (0.15 μg/kg); muscle relaxation was achieved with atracurium (0.5 mg/kg, then 0.1 mg/kg in repeated boluses *pro re nata*). Anaesthesia was maintained with sevoflurane in a N_2_O/O_2_ 2:1 mixture. Prior to surgery an epidural catheter was placed, under local anaesthesia, in accordance with the dermatomes of the operative site to administer 0.2% ropivacaine and fentanyl (5 μg/mL) in a continuous epidural infusion. Both groups received a single i.v. dose of prophylactic antibiotics, cefuroxime and metronidazole (1.5 g and 0.5 g, respectively). The levels of MIF, tumour necrosis factor (TNF-α), interleukin (IL)-lβ, IL-8, prealbumin, albumin, fibrinogen, and C-reactive protein (CRP) were preoperatively documented at 60 minutes prior to the procedures (T0), postoperatively at 60 minutes immediately after the surgery (T1), and on three consecutive postoperative days at 24, 48, and 72 hours respectively (T2, T3, T4). To evaluate disease progression and the extent of organ failure the multiple organ dysfunction score (MODS) elaborated by Marshall et al*.* (16) that considers the functional parameters of six organ systems was used. The score was determined on admission and later in the first 3 days. Performance of the respiratory system was assessed by the PaO_2_/FiO_2_ ratio, and for the cardiovascular system the pulse rate (P), the central venous pressure (CVP), and the mean arterial pressure (MAP) were monitored. Hepatic and renal functions, along with the haematopoietic system, were evaluated from the serum bilirubin, creatinine levels, and the platelet count, respectively. Central nervous system (CNS) function was assessed with the Glasgow coma scale. MODS, which ranges between normal function (0 points) and severe multiorgan failure (4–20 points), was calculated as a sum of the individual organ functions.

### Cytokine assays

Serum MIF levels were measured by an enzyme-linked immunosorbent assay (ELISA) with a Duo set ELISA development system from R&D Systems, Minneapolis, USA, according to manufacturer's instructions. Every sample was diluted in the respective ELISA assay buffer. Serum IL-1β, IL-8, and TNF-α were determined by an automated chemiluminescent immunoassay analyser, DPC (now Siemens) Immulite 1000, Deerfield, USA. For procalcitonin assays, the immunochemiluminescent kit of BRAHMS Diagnostica, Berlin, Germany was used in a Berthold LB 9507 luminometer with two programmable injectors. Albumin levels were measured by the bromo-cresol-green dye binding method (Hitachi 917, Roche), while prealbumin and α1-glycoprotein concentrations were assayed by immunoturbidimetry (Hitachi 704, OrionPharma). C-reactive protein concentrations were determined using a Beckman Immage nephelometer. Plasma fibrinogen levels were assayed by direct determination of fibrinogen (System CA 1500) using Stago thrombin reagent.

### Statistical analysis

The data are shown in median and interquartile ranges. The statistical analysis was carried out using the Statistical Program for Social Sciences for Windows (SPSS), using Mann-Whitney and Kolmogorov-Smirnov and analysis of variance (ANOVA). The correlation of the two variables was tested using Spearman's rank correlation analysis. The temporal changes of the examined parameters were analysed by the Wilcoxon test. The level of significance was set at *P* < 0.05.

## Results

The demographic characteristics of the patients and the details of the operations are summarized in Table I. The average duration of the surgical procedure was significantly longer in group B compared to that of group A. There were no differences concerning intraoperative transfusions.

**Table I.** Demographic data of patients and multiple organ dysfunction scores (MODS) in the two groups.

	Group A (*n* = 28)	Group B (*n* = 30)	*P*
Age (years)	53 (34–65)	63 (22–77)	NS
Gender (M/F)	19/11	21/7	NS
Duration of operation (min)	160 (90–270)	240 (60–440)	<0.001
Body weight (kg)	71 (54–85)	80 (63–118)	NS
Height (cm)	169 (154–183)	173 (160–194)	NS
MODS preoperatively (T0)	2 (1–3)	2 (1–3)	NS
MODS postoperatively (T1)	2 (1–3)	1 (1–3)	NS
MODS day 1 (T2)	2 (1–3)	1 (1–3)	NS
MODS day 2 (T3)	2 (1–3)	2 (1–3)	NS
MODS day 3 (T4)	2 (1–4)	3 (0–3)	NS

Our data are presented as median (interquartile range). For the statistical analyses the Mann-Whitney test was used. The duration of operation was significantly higher in group B.

### Macrophage migration inhibitory factor

**Figure 1.**
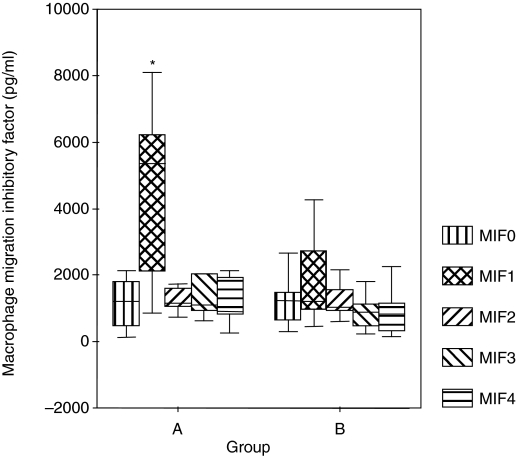
Serum MIF levels preoperatively (MIF0), immediately postoperatively (MIF1), and on the three consecutive days (MIF2, MIF3, MIF4). The data were plotted as a ‘box plot’. The minimum–maximum and interquartile ranges are shown on the figure. The difference in the two groups was verified by the Mann-Whitney *U* test. *P < 0.006.

In both groups the MIF preoperative values (MIF0) were found to be within the normal range, and there was no significant difference between the two groups. Immediately following surgery the MIF values (MIF1) measured in those patients undergoing hepatic resection were significantly higher (4,505 pg/mL) compared to the bowel resection group (1,774 pg/mL) shown in Table II. On the first postoperative day normal values of MIF were measured in both groups, and these values did not change on the second or the third postoperative days as shown in Figure 1.

**Table II.** Macrophage migration inhibitory factor (MIF) values in the two groups.

Groups	Group A	Group B	ANOVA *P*
MIF0	1137.87 ± 741.53	1462.94 ± 1276.49	NS
MIF1	4505.87 ± 551.72	1774.13 ± 1125.39	0.001
MIF2	1661.75 ± 869.88	1506.28 ± 1097.42	NS
MIF3	1558.50 ± 1113.55	1490.97 ± 1358.18	NS
MIF4	1302.33 ± 741.25	996.24 ± 913.85	NS

Values are means ± SD. For the statistical analyses the ANOVA test was used.

### Procalcitonin

On the first postoperative day, both groups showed higher procalcitonin levels (PCT2) than the values reported in literature (17). It slowly declined on subsequent days (PCT3, PCT4), but these changes were not significant compared to the initial values in these groups.

### Tumour necrosis factor

In group A the average TNF-α level was found to increase moderately but gradually; on day 3 decreased levels were observed in group A: TNF-α0: 5.1 pg/mL (4–19 pg/mL); TNF-α3: 8.5 pg/mL (4.9–9.32 pg/mL). In group B, there was also a gradual increase, with highest levels on day 3: TNF-α0: 5.1 pg/mL (4–6 pg/mL); TNF-α3: 8.9 pg/mL (7.3–16.2 pg/mL). No significant difference was found between the two groups concerning daily TNF-α levels. No correlation was observed in groups A and B between the high MIF1 and TNF-α levels.

### C-reactive protein

In the first 24 hour the CRP level was within the normal range (CRP0, CRP1), but all subsequent values fell in the pathological range (CRP2, CRP3, CRP4).

### Other parameters

In both groups a gradual decrease of albumin and prealbumin levels were observed without significant difference between groups. Similarly, no significant difference was observed regarding the IL-lβ, IL-8, α1-glycoprotein, and fibrinogen levels.

### Organ dysfunctions

Data obtained with MODS are summarized in Table I. There was no statistically significant difference between the two groups.

There was no correlation between MIF levels and early postoperative morbidity and mortality (Spearman's rho = 0.21; *P* = 0.24).

## Discussion

Sympathetically activated stress reactions might contribute to multi-organ failure in the postoperative period of major abdominal operations. In the pathogenesis of this process, pro-inflammatory cytokines (TNF-α, IL-1, IL-6) along with systemic endotoxaemia may play a pivotal role. The secretion of MIF has been shown to induce production of these critical pro-inflammatory cytokines and has been detected in several organs. The main sites of MIF production are the immune cells, i.e. monocytes, macrophage-activated T lymphocytes, and also the anterior pituitary. The most frequent causes of MIF release are pro-inflammatory stimuli, especially endotoxins, TNF-α, and interferon-γ. Systemic endotoxaemia may follow complement-driven systemic activation of the inflammatory cascade that damages the microcirculation, leading to ischaemia and reperfusion injuries, and may also emerge from bacterial translocation due to tissue hypoperfusion of the bowel wall.

In our prospective, descriptive study we assessed the induction of macrophage migration inhibitory factor levels in cancer patients undergoing bowel resection (elective enterotomy) in comparison with cancer patients undergoing liver resection (without enterotomy). Gando et al. reported the highest MIF levels at 24 hours following hepatolobectomy, and this was explained by stress-induced hypothalamo-hypophyseal activation and also by the liberation of endotoxins (13). In our study, in group A, a significantly higher level of MIF was detected immediately after the procedures that normalized on the first postoperative day. No association was observed between elevated MIF levels and early postoperative complications. There was no correlation of immediate postoperatively elevated MIF levels with the kinetics of cytokines and acute phase proteins measured in this study. It has been reported that the most effective stimulus of PCT release is bacterial endotoxin, hence similar PCT levels of the groups here might reflect comparable endotoxin exposure (17). The surgical stimulus activates the hypothalamo-hypophyseal-adrenal system, resulting in increased plasma ACTH, glucocorticoid, and MIF levels (18). With the help of immunohistochemical analysis, MIF has been expressed in hepatocytes in the areas extending out from the central veins to the portal tracts (19). Hira et al. have shown MIF expression to play a crucial role in hepatocellular carcinoma (20). Consistent with the notion, since both of our experimental groups comprised cancer patients and the preoperative MIF values did not differ, we assume that highly elevated immediately postoperative MIF levels of patients undergoing liver resection are explained by tissue damage-related release of MIF from hepatocytes rather than by inflammatory stimulus-driven secretion. This hypothesis is further supported by our findings that the operations lasted significantly longer in group B, hence we assumed that the inflammatory response is greater and the MIF secretion was significantly lower. Despite the longer operation time, the surgical insult corresponded well in the two groups, therefore a similar increase of the PCT and TNF-α levels, in accordance with previous reports including ours, could be attributed to surgical tissue damage (17,21). Our study was limited to a certain extent by the unavailability of the liver function tests, but it serves as a pilot investigation for further studies to delineate the postoperative kinetics of MIF in different surgical procedures.
